# Evaluation of Microgap Size and Microbial Microleakage at the Implant Fixture–Abutment Interface in Original and Compatible Abutments

**DOI:** 10.1155/bmri/2530986

**Published:** 2025-07-09

**Authors:** Reza Sharifi, Tahereh Ghaffari, Shima Ghasemi, Hossein Samadi Kafil

**Affiliations:** ^1^Department of Prosthodontics, Faculty of Dentistry, Tabriz University of Medical Sciences, Tabriz, Iran; ^2^Drug Applied Research Center, Department of Microbiology and Virology, Faculty of Medicine, Tabriz University of Medical Sciences, Tabriz, Iran

**Keywords:** abutment, fixture, implant, microgap, microleakage

## Abstract

**Introduction:** The microgap between implant and abutment can lead to mechanical and biological problems such as abutment screw breakage or peri-implantitis. The utilization of compatible abutments in place of the original components results in a decrease in costs and provides dentists with a broader selection of abutment options. The purpose of this study was to compare bacterial microleakage and microgap in the Korean Osstem, Dio, and Cis systems, whose regular abutments are compatible with each other.

**Method:** Twelve implant abutments were used in this study, each four from Osstem (Seoul, South Korea), Dio (Seoul, South Korea), and Cis (Medimecca, Seoul, South Korea), all measuring 7 mm in length, 4.5 mm in diameter, and 3 mm in height. These were attached to Osstem fixtures of regular size (4.5 mm diameter and 11.5 mm length), which were contaminated with a bacterial suspension. Bacterial contamination was assessed at 5, 24, and 48 h, while microgap size was measured at four points using an electron microscope. Statistical analyses, including one-way ANOVA and the Kruskal–Wallis tests, were performed by SPSS 17 to evaluate differences in microgap and microleakage among the groups.

**Results:** There is a statistically significant difference between the mean microgap in the Dio group and the other two groups. The mean microgap in the Dio group was higher than both of the other groups (*p* value < 0.001). There is no statistically significant difference between the mean microgap in the Osstem group and its mean in the Cis group (*p* value > 0.05). There was no statistically significant difference between the mean number of colonies (CFU) in the study groups at any of the four evaluation times (*p* value > 0.05).

**Conclusion:** Considering the limitations of this study, it can be inferred that the Cis-compatible abutment is also applicable for use with the Osstem fixtures.

## 1. Introduction

Currently, the use of osseointegrated dental implants is widely regarded as the preferred solution for replacing missing teeth among dental professionals. This treatment modality has demonstrated strong long-term reliability, with studies indicating survival rates of approximately 94.5% after 5 years and 89.4% after 10 years [1]. Despite these promising outcomes, certain complications have been noted [2]. One notable concern is the formation of a microgap at the interface between the implant and the abutment after their connection. These microgaps are typically found along the screw threads and at the base of the screw, providing a potential entry point for bacterial invasion.

The structural integrity at the implant–abutment interface plays a crucial role in several aspects of implant success. It aids in minimizing stress transmission to the surrounding bone, maintaining the mechanical stability of the abutment screw, and preventing micromovements at the junction. The type of connection between the implant and abutment greatly influences the joint's resistance to torque and lateral forces. Early external hexagonal connection systems, for example, offer limited stability against lateral loading and torsional forces, with reports showing screw loosening rates ranging from 6% to as high as 48%. As a result, internal connection geometries are now more widely adopted, as they offer deeper load distribution and provide better protection for the screws by reducing excessive stress, thereby enhancing the overall strength and durability of the connection [3].

Research has established that even minimal microgaps can allow for bacterial infiltration, which may contribute to peri-implantitis [4]. The accumulation of bacteria at this interface is considered a major factor in the initiation of soft tissue inflammation and subsequent degradation around the implant [5]. These findings emphasize the need for precise fitting and minimizing microgaps during implant restoration to reduce the risk of infection.

Several factors influence the extent of the microscopic gap and associated microleakage at the implant–abutment interface [6–8]. These include the specific implant system employed, the structural design of the implant–abutment connection, the choice between cast and machined abutments, the torque applied during abutment installation, and whether a screw-retained or cement-retained abutment is used [9]. Various interface geometries—such as conical, platform-switching, and screw-type connections—present distinct characteristics that affect both microgap dimensions and the mechanical integrity of the connection [3].

In instances where original manufacturer abutments are either inaccessible or cost-prohibitive, clinicians may resort to third-party compatible abutments. However, when using such alternatives, it is critical that the interface design closely matches the intended implant system to avoid excessive discrepancies. Gaps exceeding 10 *μ*m are particularly problematic, as they can facilitate bacterial ingress. Optimally engineered abutment connections contribute significantly to the longevity and clinical success of implant-supported restorations [4, 5, 7–10].

Considering that certain brands of dental equipment are popular and more commonly used in each country, and no study has been conducted on the microgap and microleakage of common Korean implant systems, the purpose of this study was to evaluate the microgap and microbial microleakage of the Osstem implant system connected to the original and compatible abutment (Dio and Cis systems).

## 2. Methods and Materials

### 2.1. Sample Size

To determine the sample size, we referred to the study conducted by Berberi et al. [11]. Considering a Type I error of 5% and a statistical power of 90%, the initial calculation yielded a requirement of three samples per group. To enhance the validity of the study, we increased the sample size by 20%, resulting in a final selection of four samples per group.

In this study, the independent variable is the type of abutment used (Osstem, Dio, or Cis), while the dependent variables include microgap size and bacterial microleakage. Descriptive statistics, including mean and standard deviation, were provided for each variable, and 95% confidence intervals (CIs) have now been included to better represent the variability in the data.

This study was conducted on 12 implants (Osstem Implant, Seoul, South Korea) with the same size (regular, diameter 4.5, length 11.5 mm), which were divided into three groups, and each group included four implants. Three types of abutments that are compatible with the Osstem system and whose internal connections are similar (Morse taper and internal hex connection) were used, and all the components were prefabricated. Four Osstem abutments (Seoul, South Korea), four Dio abutments (Seoul, South Korea), and four Cis abutments (Medimecca, Seoul, South Korea) all regular size with length = 7, diameter = 4.5, and height = 3 mm were used.

The compatible abutments used in this study were manufactured in Korea; however, they were produced by different manufacturers. While all abutments were designed to fit the Osstem implant system, variations in manufacturing processes and tolerances may influence the microgap and microbial leakage. This aspect is a key point of investigation in our study.

The difference in torque values (5 Ncm) was due to the manufacturers' recommended torque specifications for each abutment system. While a 5 Ncm difference is relatively small, it may influence the adaptation of the abutment–implant interface, potentially affecting the microgap size and bacterial leakage. However, this variation reflects real clinical scenarios where different manufacturers provide specific torque recommendations for their components. To account for this factor, we ensured that each abutment was torqued according to the manufacturer's guidelines, maintaining consistency within each group.

These abutments are manufactured with specific design features to ensure compatibility, such as the conical geometry and the inclination of the abutment–implant connection, which were consistent across all systems. The inclination of the abutment cone-in-cone profile and the length of the contact area were also measured and taken into consideration, as these factors can influence microgap size and bacterial infiltration. Additionally, the metal alloys used for both the abutments and implant fixtures were specified. The abutments and fixtures were made of titanium alloys, but the alloys used for each system may differ slightly in terms of mechanical properties (e.g., compressive and torsional strength) due to different industrial processes involved in their production.

### 2.2. Contamination of Samples and Microbiological Evaluation

To perform a microbial microleakage test, the implant fixtures along with 12 abutments were autoclaved under standard conditions (15-Pa pressure and 121°C temperature). Also, all the tools used in this process were sterilized [12]. To perform the microbial test, *Escherichia coli* (*E. coli*) bacteria, which are gram-negative, motile, facultative anaerobic bacteria, with 1.1–1.5 *μ*m diameter and 2–6 *μ*m length, were used [13]. This bacterium has been widely used in similar studies [12].

Pure culture medium (*E. coli* ATCC 25922) was used for biological purposes. Bacterial suspensions were prepared by culturing microorganisms on blood agar (Mark Darmstadt, Germany) and incubating them for 24 h. They were diluted in tryptic soy broth (TSB) (TSB Difco, Lawrence, Kan) to reach McFarland's 0.5% standard (1.5 × 10^8^ CFU/mL). A volume of 0.5 *μ*L of bacterial suspension were incubated by fine micropipettes on the inner surface of each implant. Then, Osstem group abutments with a torque of 30 Ncm and Cis and Dio abutments with a torque of 35 Ncm were connected to the implants according to the torque recommended by the manufacturer. To ensure that the external surface of the implant–abutment assembly was not contaminated during operation, sterile conical paper covers were placed on the surface and sampling was done. Each of the sterile paper covers was submerged in 5 mL of nutrient broth and incubated at 37°C to determine surface contamination during operation. If positive, the sample was sterilized, and the incubation steps were repeated.

All samples were placed in Eppendorf microtubes, and the TSB culture medium was added to the point that the level of the culture medium was lower than the access space to the abutment. A volume of 50 *μ*L of culture medium was changed around each implant every 12 h. Then, all the samples were transferred to the incubator, and after 5, 24, and 48 h and 14 days, 0.1 mL of the culture medium around the implants was removed by the sampler and cultured on Mueller Hinton agar plates [14]. In these conditions, the number of bacteria may be uncountable; therefore, 0.1 mL of the culture medium from each sample was removed and cultured on the plate with a dilution of 1–10.

In cases where bacterial microleakage occurred between the implant and the abutment, colonies were visible and countable on the plate. Then, based on the number of colonies counted on the culture medium, the number of colonies in 1 mL of culture medium around the implant at a specific time was calculated (CFU per milliliter). To ensure the presence of *E. coli* on the plate, eosin methylene blue (EMB) biochemical tests were performed [15].

### 2.3. Evaluation of Microgap Using Electron Microscope

After the microbiological evaluations, all the samples were placed in the ultrasonic device and then sterilized in the autoclave [13]. Then, the abutments were connected to the fixtures with the torque recommended by the manufacturer, and the microgap in each sample was examined using an electron microscope (Tescan MIRA3 FEG-SEM, Kohoutovice, Czech Republic). The size of the microgap was measured at four randomly selected points on each sample, and the ratios were determined by comparing them with the numbers on the scale bar [13].

### 2.4. Statistical Analysis

The results were reported as the mean ± standard deviation and number (percentage). One-way analysis of variance and the Kruskal–Wallis test were used to compare microgap and microleakage between the studied groups. SPSS Version 17 was used for data analysis. A probability value of less than 5% was considered a significant level. The dependent variables included microgap size and bacterial leakage, while the independent variables are the type of abutment and the implant system used.

The study was approved by the regional ethics committee (IR.TBZMED.VCR.REC.1401.289).

## 3. Results

**Figure 1:**
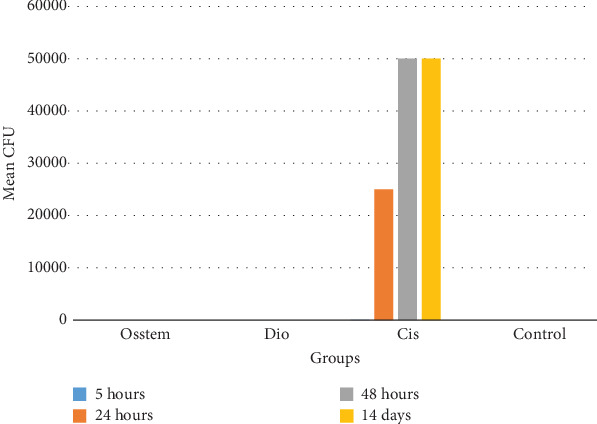
Column chart of mean CFU in the study groups and evaluation times.

The mean and standard deviation of the CFU in the four groups and the four evaluation times are shown in Figure 1. The mean CFU was greater than 0 only in the Cis group, and in this group, the mean CFU was higher at two times of 48 h and 14 days than at the other two evaluation times.

The normality of the distribution of CFU variable was assessed by the Kolmogorov–Smirnov test. The results showed that, in all four evaluation times, the distribution of the CFU variable was not normal (*p* value < 0.001); therefore, a nonparametric Kruskal–Wallis test was used. The results of this test are given in Table 1. In any of the four evaluation times, there is no statistically significant difference between the mean rank of the CFU in the study groups (*p* value > 0.05).

**Figure 2:**
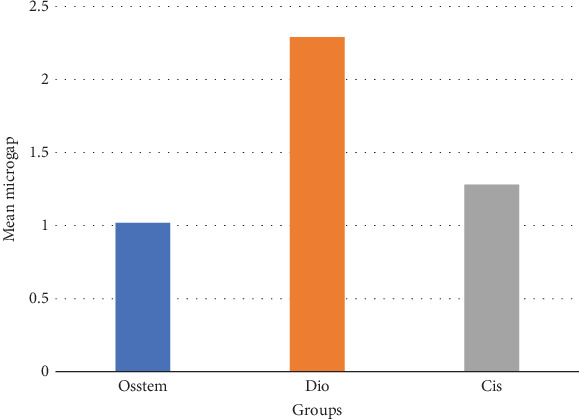
Column chart of the mean microgap in study groups.

The mean and standard deviation of the microgap in the groups are given in Figure 2. The normality of the distribution of the microgap was assessed by the Kolmogorov–Smirnov test. The results showed that the distribution of the microgap is normal (*p* value = 0.118); therefore, a parametric test, one-way ANOVA, was used. The results of this test are given in Table 2.

Table 2 shows that there is a statistically significant difference between the mean microgap in the study groups (*p* value < 0.001). To evaluate the homogeneity of the variance of the groups, Levene's test was used, and it was found that there was no statistically significant difference between the variances of the study groups (*p* value = 0.053); therefore, Tukey's post hoc test was used. The results of this test are given in Table 3.

**Figure 3:**
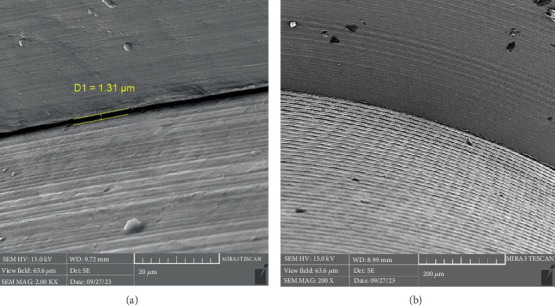
The microgap observed in the electron microscope related to the Dio system at (a) x20 and (b) x 200 magnification.

**Figure 4:**
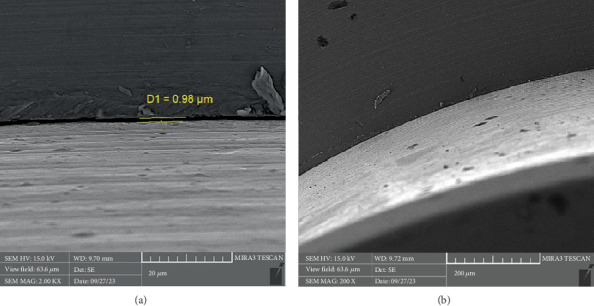
The microgap observed in the electron microscope related to the Cis system at (a) x20 and (b) x200 magnification.

**Figure 5:**
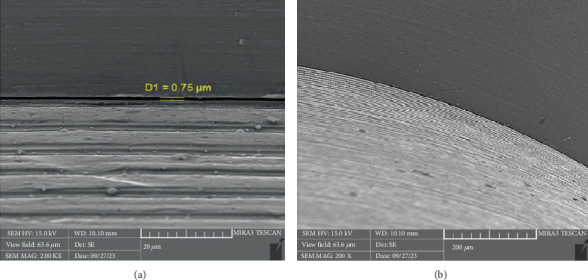
The microgap observed in the electron microscope related to the Osstem system at (a) x20 and (b) x200 magnification.

There is a statistically significant difference between the mean microgap in the Dio group (Figure 3) and the other two groups. The mean microgap in the Dio group was higher than both of the other groups (*p* value < 0.001). There is no statistically significant difference between the mean microgap in the Osstem group (Figure 4) and its mean in the Cis group (Figure 5) (*p* value > 0.05***).***

## 4. Discussion

The interface between dental implants and abutments inherently possesses a microgap, which can serve as a niche for bacterial colonization. This gap arises due to the inherent limitations in manufacturing precision, making a perfect fit between the two components challenging. As the microgap widens, the potential for microleakage escalates, facilitating bacterial ingress that may lead to inflammation in the peri-implant tissues, especially given the proximity to the alveolar bone crest and surrounding soft tissues [16, 17].

Prolonged bacterial presence at this junction can stimulate the release of proinflammatory cytokines and promote osteoclast activity, potentially resulting in bone resorption. Mechanical loading, such as occlusal forces, can exacerbate this issue by encouraging bacterial colonization at the implant–abutment interface, thereby increasing the risk of peri-implantitis and marginal bone loss. Utilizing abutments not originally designed for the specific implant system may further amplify microgap dimensions and micromovements, hence the recommendation to employ original manufacturer abutments [18, 19].

In this study, titanium implants featuring conical connections were selected due to their favorable marginal fit and widespread clinical use. To assess compatibility, machined abutments from different manufacturers were compared. Given the lack of prior comparative analyses of these specific systems, the study was aimed at evaluating the microgap and microbial leakage at the implant–abutment interface in Korean implant systems using both original and third-party abutments.

The formation of microgaps can be influenced by various mechanical factors, including the connection type, geometric design, material composition, and surface treatment of the implant–abutment assembly. Considering that bacterial cells are typically less than 2 *μ*m in size, even minimal gaps can permit bacterial adhesion and colonization. However, microgaps smaller than 10 *μ*m are generally deemed clinically acceptable due to their negligible biological and mechanical impact [20]. In this investigation, the average microgap measured at the implant–abutment interface was 1.02 *μ*m for original Osstem abutments, 1.28 *μ*m for Cis-compatible abutments, and 2.29 *μ*m for Dio-compatible abutments, all falling within acceptable clinical thresholds.

Previous research by Zanardi et al. compared the SIN, Conexão, and Neodent systems, finding that the Conexão system exhibited larger microgaps than the other two. Additionally, the use of third-party abutments increased the microgap in the SIN and Neodent systems. Their study reported an average microgap of 4.6 *μ*m, whereas the current study observed a mean of 1.53 *μ*m, likely attributable to differences in implant systems and connection designs evaluated [21].

Kowalski et al. examined the Apollo and Astra abutments, noting that the Apollo abutments had larger microgap volumes compared to Astra. Despite this, the linear and volumetric dimensions of the microgaps were similar, and all were within clinically acceptable limits [16].

Berberi et al. assessed the Tech Tx Astra implant system with four abutment types—TiDesign, Dual, Natea Plus, and Implanet—all sharing the same internal conical interface. Their findings indicated superior external and internal fit when original components were used [22]. Similarly, a systematic review by Tallarico et al. concluded that original and certified nonoriginal abutments outperformed compatible abutments regarding mechanical properties, microleakage, and marginal adaptation [23].

Duraisamy et al. reported significant differences in microgap sizes between original and nonoriginal abutments, with the latter exhibiting larger gaps. Nonetheless, the mean microgap at the implant–abutment junction for both groups remained within clinically acceptable limits. Berberi et al. also demonstrated that using compatible abutments led to increased micromovements compared to original abutments, emphasizing the importance of component compatibility [11].

Based on the results of the present study and similar studies, it seems that the microgap between the implant and the abutment depends on the brand, and each brand's compatibility should be evaluated before use. It should also be considered that the distance between the implant and the abutment is a three-dimensional space and not a line, and the linear measurement of the microgap may not be completely accurate.

Microbial leakage evaluation methods are categorized into internal and external approaches. The internal method assesses leakage from the implant's exterior to its interior, simulating in vivo conditions but requiring disassembly of the implant–abutment assembly, which can introduce contamination and limit repeatability [24–27]. Conversely, the external method evaluates leakage from the interior to the exterior, allowing for repeated measurements over time [28]. In this study, the external method was employed using *E. coli*, a Gram-negative, facultative anaerobe approximately 1.1–1.5 *μ*m in diameter, commonly used in in vitro studies due to its well-documented colonization capabilities [13, 29].

In the present study, there was no statistically significant difference between the mean CFU in the study groups at any of the four evaluation times. Contrary to our study, Berberi et al.'s study, which used the osseospeed implant system with original and compatible abutments Implanet, TiDesign, Dual and Natea, showed that microleakage was significantly lower in the TiDesign and Dual groups [30]. Rhodamine B (RhB) was used in this study. RhB is used as a fluorescent dye for biological assays. RhB can pass through gaps faster than endotoxin (50–100 kDa) and bacteria (1.1–1.5 *μ*m). These findings may explain why leakage rates were higher than the present study.

Other studies have indicated that bacterial microleakage increases over time; however, this study did not observe temporal differences, possibly due to variations in implant systems and measurement methodologies. Rismanchian et al. investigated four abutment types—Cast-On, Castable, Solid, and Synocta—connected to Straumann fixtures, finding significant microleakage within the first 5 h but not at later time points. Their results align with the current study, suggesting that well-adapted abutment–fixture assemblies can prevent microleakage over extended periods [25, 27, 31].

In the study of Rismanchian et al., four groups of Cast-On, Castable, Solid, and Synocta abutments connected to Straumann fixtures were investigated. The mean number of leaked colonies (CFU per milliliter) in the implant and abutment junction was significant in the first 5 h. However, no significant difference was seen at 24 h, 48 h, and 14 days. The use of Solid and Synocta abutments can significantly reduce the size of the microgap. However, Cast-On abutments do not show significant differences with Castable abutments in terms of microgap. They showed that the use of Solid abutments instead of Synocta and vice versa did not have a significant effect on the microgap. The use of premachined titanium abutments (Solid and Synocta) can reduce the distance between implants and abutments compared to Cast-On and Castable abutments [13]. In terms of the effect of time on bacterial microleakage, the results of this study were different from our study. The reason for this difference can be due to the difference in the type of implants and the used torque. However, in Rismanchian et al., similar to the present study, microleakage did not occur until the 14th day in two samples (Solid and Synocta). This can indicate that if the abutment and the fixture have adaptation, the microleakage can be zero.

Khajavi et al. compared Zimmer systems with slip-joint connections to Argon systems with conical joints, concluding that connection type significantly influences bacterial leakage, though leakage did not vary significantly over time [28]. Siadat et al. observed increased microleakage in all abutment types after cyclic loading, with GoldAdapt abutments exhibiting the least leakage. Their use of radiotracers, which offer higher accuracy, may account for differences in findings compared to the current study [32].

Variability in microleakage results across different implant systems and studies may stem from differences in bacterial inoculation volumes, measurement techniques, and the use of dyes versus microbial assessments. For instance, da Silva-Neto et al. and Kano et al. found no difference in vertical misfit between Cast-On and cast abutments made from different alloys, highlighting the importance of evaluating both horizontal and vertical compatibility [33, 34].

In the present study, bacterial microleakage was observed in one of the samples of the Cis group, but none in the rest of the samples, which is probably related to the manufacturing error in the abutment or fixture [35]. Since only one leakage point is enough for bacterial microleakage, and the microgap evaluation was conducted randomly in four points, the point of leakage may not have been observed in the electron microscope images.

Mechanical loading can also influence bacterial colonization in microgaps. Steinebrunner et al. reported significant differences among five implant systems concerning chewing cycles and bacterial colonization. The absence of loading in the current study may explain the lack of significant differences in microleakage among implant systems over time [26].

The study concluded that the Dio group exhibited a higher mean microgap compared to the Osstem and Cis groups. However, since all measurements fell within clinically acceptable ranges, both Cis and Dio abutments may be considered suitable for use with Osstem implants. Variations in study outcomes regarding system compatibility may result from the lack of standardized methods, heterogeneity among implant systems, and differences in microgap and microleakage evaluation techniques. It is important to note that this study did not incorporate cyclic loading or fatigue tests, which may affect the applicability of results to clinical conditions. Additionally, the presence of saliva in the oral environment could influence outcomes compared to in vitro studies conducted in dry settings.

## 5. Limitations of the Study

Limitations of this study include a relatively small sample size, the use of a single bacterial strain, and potential inconsistencies in the torque applied to abutments, all of which may impact microgap and bacterial leakage measurements. Despite these constraints, the study offers valuable insights into the compatibility and bacterial leakage associated with different implant systems. Further research with larger sample sizes and a broader range of bacterial strains is necessary to validate these findings.

## 6. Conclusion

Considering the limitations of this study, it can be inferred that Cis-compatible abutments are suitable for use with Osstem fixtures. It is recommended that a similar study be conducted using cyclic loading. Additionally, comparing the compatibility of other brands with different types of abutments may provide valuable insights. Conducting clinical studies and a large sample size could also contribute significantly to elucidating this matter. Future studies should focus on further refining implant and abutment designs to reduce the microgap, as well as testing the effects of different bacterial strains and clinical scenarios. The present study provides a foundation for understanding the role of the microgap in peri-implantitis and highlights the importance of selecting compatible implant components to ensure optimal clinical outcomes.

## Figures and Tables

**Table 1:** Mean microleakage in the study groups at four times.

**Time**	**Group**	**Mean microleakage**	**p** ** value**⁣^∗^
5 h	Osstem	8.00	0.392
	Dio	8.00	
	Cis	10.00	
	Control	8.00	

24 h	Osstem	8.00	0.392
	Dio	8.00	
	Cis	10.00	
	Control	8.00	

48 h	Osstem	8.00	0.392
	Dio	8.00	
	Cis	10.00	
	Control	8.00	

14 days	Osstem	8.00	0.392
	Dio	8.00	
	Cis	10.00	
	Control	8.00	

⁣^∗^*p* value based on the Kruskal–Wallis test.

**Table 2:** One-way analysis of variance test results for microgap.

**Source of variation**	**Sum of squares**	**Degree of freedom**	**Mean squares**	**F** ** value**	**p** ** value**
Between groups	14.480	2	7.240	37.543	< 0.001
Within groups	8.678	45	0.193		
Total	23.158	47			

**Table 3:** Results of the comparison of study groups for microgap with Tukey's test.

**Group (I)**	**Group (J)**	**Mean difference (I–J)**	**p** ** value**
Osstem	Dio	1.27-	< 0.001
	Cis	−0.26	0.212
Dio	Cis	1.01	< 0.001

## Data Availability

The data that support the findings of this study are available on request from the corresponding author. The data are not publicly available due to privacy or ethical restrictions.
